# PReS-FINAL-2257: Pediatric rheumatology in a rare disease working group: examples of diagnoses

**DOI:** 10.1186/1546-0096-11-S2-P247

**Published:** 2013-12-05

**Authors:** A Solyom, B Mosdósi, Z Nyul, K Komlósi

**Affiliations:** 1 Pediatric Hospital, University of Pécs, Pécs, Hungary; 2 Medical Genetics Department, University of Pécs, Pécs, Hungary

## Introduction

As part of an initiative to expand diagnostic resources, information, and quality of care for patients with rare diseases in Hungary, a network of specialists dealing with rare diseases was established at our University Clinical Center, and a Rare Disease Working Group was formed within the Pediatric Hospital.

## Objectives

We hope to document a selection of diagnoses, which came about through close cooperation between the pediatric rheumatology and clinical genetics services at our Pediatric Hospital, in the context of an initiative to better evaluate and care for patients with rare diseases, or who present a differential diagnostic dilemma.

## Methods

Each of the following cases were seen together by a clinical geneticist and pediatric rheumatology service physician involved in the Rare Disease Working Group. We document here three different cases from among those seen with complaints and presentations representing part of the pediatric rheumatology disease spectrum.

## Results

Case 1: A 14 year old male presented with painless swelling of PIP joints II-V on both hands which had been consistently present for over one year. The patient was seen in a joint session (clinical genetics, rheumatology service), and the diagnosis of pachydermodactyly established, sparing the patient further diagnostic interventions and unnecessary treatments.

Case 2: A 6 year old boy presented initially at 3 years of age with bilateral coxitis. One month later, significant radiological changes in both hips (including the acetabulum) were noted, with relatively minor physical complaints. He was initially diagnosed with Legg-Calvé-Perthes Disease by an orthopedic surgeon. Through our clinic, urine glycosaminoglycan, enzymatic and genetic testing confirmed the diagnosis of mucopolysaccharidosis II (Hunter Disease). In addition to the initiation of enzyme replacement therapy, the diagnosis allows for planning of specialized care, follow up and intervention with regard to the patient's progressive joint disease.

Case 3: An 8 year old girl had been seen by a pediatric neurologist and orthopedic surgeon because of symmetrical painless contractures of her hips, knees, ankles and the small joints of her feet, which were increasingly affecting her everyday activities. She was referred to our clinical geneticist because of suspicion of a lysosomal storage disease. Upon examination the patient had scleroderma-like lesions symmetrically affecting both lower limbs. Findings on laboratory, imaging, and histopathological examination led to the tentative diagnosis of congenital fascial dystrophy, or "Stiff-skin Syndrome". An analysis of the Fibrillin-1 (FBN1) gene, recently found to be mutated in several such patients, found no alterations. We are closely following the patient, and additional extensive genetic analysis will hopefully be initiated in the near future.

## Conclusion

We hope to encourage the consideration of these diseases (albeit rare) as potential differential diagnoses, and the establishment of such "rare disease working groups" where they may not yet exist.

## Disclosure of interest

None declared.

